# Utilizing intratumoral and peritumoral ultrasound radiomics for predicting *KRAS* gene mutation status in rectal cancer patients

**DOI:** 10.1002/acm2.70153

**Published:** 2025-07-13

**Authors:** Yajiao Gan, Qiping Hu, Qingling Shen, Qingfu Qian, Peng Lin, Minling Zhuo, Ensheng Xue, Zhikui Chen

**Affiliations:** ^1^ Department of Ultrasound Fujian Medical University Union Hospital Fuzhou Fujian China

**Keywords:** endorectal ultrasound, KRAS mutation, rectal cancer, ultrasound radiomics

## Abstract

**BACKGROUND:**

*KRAS* mutations are associated with treatment and prognostic outcomes in colorectal cancer patients.There have been no studies on utilizing the peritumoral images to predict *KRAS* mutation status in rectal cancer patients. We aim to develop a radiomics model utilizing intratumoral and peritumoral ultrasound images for predicting *KRAS* mutation status in rectal cancer.

**METHODS:**

This study retrospectively included 278 patients with pathologically confirmed rectal cancer following surgery, who were randomly divided into training group (194 cases) and test group (84 cases) at a 7:3 ratio. Radiomic features from both intratumoral and peritumoral regions were extracted from endorectal ultrasound images. A five‐step procedure was used to select robust features. Based on these, intratumoral, peritumoral, and combined models were created using logistic regression, support vector machine, and light gradient boosting machine. The predictive accuracy, calibration, and clinical utility of the models were evaluated using receiver operating characteristic curves, calibration curves, and decision curve analysis. The SHapley Additive exPlanation (SHAP) method was used to evaluate the importance of the features in the optimal models.

**RESULTS:**

In the test set, the area under the curve of all three combined models exceeded that of the individual intratumoral and peritumoral models. According to area under the curve, decision curve analysis, and calibration curves, combine_LR demonstrated the best performance, with an area under the curve of 0.881.

**CONCLUSIONS:**

The ultrasound radiomics model, incorporating both intratumoral and peritumoral features, effectively predicts *KRAS* status in rectal cancer patients, potentially guiding clinical targeted therapy selection.

## 1 INTRODUCTION

Colorectal cancer ranks as the second leading cause of cancer‐related deaths globally, with rectal cancer constituting over one‐third of all colorectal cancer cases.^1^, ^2^, ^3^ Antiepidermal growth factor receptor (EGFR) drugs, such as paroxetine and cetuximab, effectively inhibit tumor growth and proliferation by blocking the RAS signal transduction pathway, improving the prognosis of patients with metastatic colorectal cancer.^4^ However, approximately 40% of rectal cancer patients possess *KRAS* mutations,^5^, ^6^, ^7^ which activate the RAS membrane receptor tyrosine protein kinase signaling pathway, disrupt upstream signal regulation, and promote tumor cell proliferation. Consequently, these patients do not benefit from EGFR inhibitor therapy.^8^ *KRAS* mutations have also been associated with poorer 3‐year (79.9%) and 5‐year (56.7%) survival rates compared to *KRAS*‐negative patients with rectal cancer.^9^ Further, *KRAS* mutations are associated with an increased risk of relapse and mortality in patients with colorectal cancer^10^ and typically confer higher resistance to chemotherapy.

New therapies targeting the *KRAS* mutation have been investigated to optimize treatment for this subgroup of rectal cancer. Therefore, early detection of *KRAS* mutation status in rectal cancer is conducive to the choice of clinical therapy. Since 2016, the National Comprehensive Cancer Network (NCCN) guidelines have recommended genotyping for *KRAS* mutations in tumor tissues for all patients with suspected or confirmed metastatic colorectal cancer, and that patients with *KRAS* mutations should not be treated with either cetuximab or panitumumab, either alone or in combination with other anticancer agents.^4^, ^8^

Currently, tumor histopathological examination remains the gold standard for detecting *KRAS* mutations. However, many patients with rectal cancer are diagnosed at an advanced stage, having missed the opportunity for direct surgery. Colonoscopy biopsies, which yield only a small amount of tissue, may not fully determine the tumor's genetic status.^11^, ^12^ Furthermore, genetic testing is both time‐consuming and expensive. Thus, finding a simpler, more economical, and effective method to predict *KRAS* status is crucial.

Radiomics, which extracts data features from images through data mining, provides a plethora of objective and quantitative data regarding tumor heterogeneity. This approach allows for various statistical analyses, including tumor diagnosis and differentiation, classification, biological risk prediction, survival prediction, and more.^13^, ^14^ These noninvasive prediction models offer a solid foundation for clinical application. Previous studies have demonstrated that radiomics features derived from CT, MRI, and PET/CT images effectively predict *KRAS* mutations in rectal cancer.^15^ However, most research has focused primarily on the primary tumor region, with limited attention given to the peritumoral environment. In fact, as the leading edge of tumor spread and invasion, the peritumoral region may contain significant biological information within its imaging features.^16^, ^17^, ^18^ Previous studies have shown that the peritumoral region of rectal cancer holds valuable information, such as for assessing preoperative prognostic factors in resectable rectal cancer.^19^

Endorectal ultrasound (ERUS) is a commonly used imaging method for rectal cancer, valuable in both diagnosis and staging.^16^, ^20^, ^21^ Several studies have investigated the potential of ultrasound as a noninvasive method to assess tumor biology and genetic mutations.^22^, ^23^ Although previous studies utilized expanded peritumoral regions (10, 20, and 30 pixels) combined with radiomics and deep learning features to predict *KRAS* mutation status in rectal cancer via ERUS imaging, the impact of a fixed physical peritumoral width remains unclear. Additionally, subgroup analyses were not conducted in these studies to evaluate the generalizability of the models across diverse patient populations. Prior research has indicated that deep learning typically achieves higher specificity compared to radiomics, with minimal differences in sensitivity and AUC between the two approaches.^15^ However, deep learning requires larger datasets; therefore, we selected radiomics for our study. Consequently, this study aimed to construct ultrasound‐based radiomics models using classical machine learning algorithms, compare the predictive value of intratumoral versus peritumoral features, and perform subgroup analyses to evaluate model applicability across diverse patient populations.

## 2 MATERIALS AND METHODS

### 2.1 Study subjects

This single‐institution retrospective study was approved by our institution and informed consent from the subjects was not required. This study collected clinical and imaging data from rectal cancer patients between June 2019 and August 2023. The inclusion criteria were as follows: (1) patients who had not received preoperative radiotherapy or chemotherapy; (2) postoperative pathological confirmation of complete T‐stage with reported *KRAS* status (wild‐type/mutant); (3) a reasonable interval between *KRAS* testing in postoperative pathological specimens and ERUS examination; and (4) high‐quality ultrasound images. The exclusion criteria were as follows: (1) patients who had received preoperative radiotherapy or chemotherapy; (2) incomplete clinical and postoperative pathological data; (3) poor image quality that did not show the complete lesion; and (4) an interval exceeding 30 days between postoperative pathological specimen *KRAS* testing and ERUS examination. A total of 278 patients meeting these criteria were randomly divided into a training set (*n* = 194) and test set (*n* = 84) at a 7:3 ratio.

### 2.2 ERUS examination and image acquisition

The examination was conducted using a HITACHI Preirus color Doppler ultrasound diagnostic system. Two types of probes were utilized: an endocavitary radial scanning probe (model: R54AW‐19, frequency range: 5–10 MHz) and an intracavity end‐scanning probe (model: EUP‐V53W, frequency range: 4–8 MHz). Prior to the examination, patients underwent an enema to cleanse the bowel. They were then positioned in left hip and knee flexion. To create an acoustic window, 150 mL of warm water was injected into the anus. A disposable isolation cover was placed over the probe, which was then gently inserted into the rectum for systematic and comprehensive scanning. We meticulously recorded the primary characteristics of the lesions, including their maximum diameter, thickness, distance from the anal verge, and the presence of enlarged lymph nodes.

### 2.3 Region‐of‐interest (ROI) segmentation and feature extraction

**FIGURE 1:**
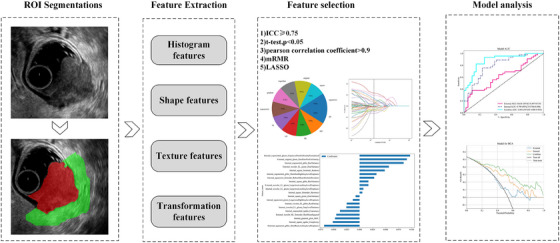
Workflow of the development of the predictive models.

The ITK‐SNAP software (version 3.8.0, http://www.itksnap.org/) was utilized for image segmentation. An experienced ultrasound doctor, Doctor A, with 10 years of diagnostic experience, selected sonograms with clear imaging and the deepest tumor infiltration. Doctor A then manually segmented the ROI. After a period of 10 days, the ROI was delineated once more by Doctor A and another ultrasound doctor, Doctor B, who has 5 years of experience in the field. The software was employed to automatically draw the peritumoral area (an expansion of 5 mm), which was subsequently fine‐tuned by Doctor A manually. It is important to note that both physicians were unaware of the patients' pathological diagnoses. We utilized the Pyradiomics library in the Python (version 3.7.0; http://www.python.org) to extract the intratumoral and peritumoral radiomics features, including first‐order statistics, shape‐based features, and texture features (GLCM, GLRLM, GLSZM, and NGTDM) from both original and wavelet‐filtered images. Figure 1 illustrates the mask dilation process and the resulting masks that represent peritumoral regions.

### 2.4 Feature selection and model construction

We developed a five‐step procedure for dimensionality reduction and robust feature selection. First, Doctor A and Doctor B independently segmented ROI. Both doctors were aware of the rectal cancer diagnosis but were not informed of the clinical and pathological specifics. The intraclass and interclass correlation coefficients (ICCs) were calculated by randomly selecting 60 ERUS images. We assessed the reliability and repeatability of the features, opting for radiomic features with an ICC > 0.75 (indicating excellent stability) for feature extraction.^24^ Second, we employed the z‐score standardization method to normalize all feature lines. Through *t*‐tests, features with *p* < 0.05 were retained. Third, we calculated the correlation between features using the Spearman correlation coefficient. For features with a correlation coefficient > 0.9, one of the two correlated features was selected. Fourth, the Max‐Relevance and Min‐Redundancy (mRMR) algorithm was used to retain the 30 features with the highest relevance. Finally, we used the least absolute shrinkage and selection operator (LASSO) logistic regression algorithm to adjust penalty parameters through 10‐fold cross‐validation. This method was then applied to select relevant features of *KRAS* status with nonzero coefficients from the training group. We utilized machine learning algorithms including logistic regression (LR), support vector machine (SVM), and light gradient boosting machine (LightGBM) to construct intratumoral, peritumoral, and fusion models, resulting in a total of nine radiomics models. To ensure model robustness and prevent overfitting, a five‐fold cross‐validation strategy was employed on the training set to select stable radiomic features and determine the optimal classifier for each model type. LR and SVM models were implemented using default parameters from the Scikit‐learn library. For LightGBM, optimal parameters were determined using grid search with five‐fold cross‐validation. The diagnostic accuracy, sensitivity, positive prediction value (PPV), negative prediction value (NPV), and specificity of the nine models were evaluated using a confusion matrix and its derived diagnostic indicators. Receiver operating characteristic (ROC) curves and decision curve analysis (DCA) were drawn to assess the predictive performance and clinical value of the models. Calibration curves and the Hosmer–Lemeshow test were used to evaluate model fit. The DeLong test was employed to compare the efficiency differences among the models.

### 2.5 Assessment of model generalizability

To evaluate the generalizability of our model across heterogeneous populations, subgroup analyses were performed based on clinical variables that significantly differed between *KRAS* wild‐type and mutant groups.

### 2.6 Interpretability of machine learning models

After determining the optimal intratumoral, peritumoral, and fusion models separately, we employed the SHapley Additive exPlanation (SHAP) method to gain a deeper understanding of feature importance, emphasizing the most influential variables. We ranked the SHAP values of the features in descending order to identify key predictive factors in our study cohort.

### 2.7 Statistical analysis

Independent *t*‐tests or Mann–Whitney *U* tests were used to analyze continuous variables in clinical data, while Fisher's exact test or chi‐square test was used for categorical variables. A two‐tailed *p*‐value of < 0.05 was considered statistically significant for identifying differences.

## 3 RESULTS

### 3.1 Baseline data of patients

This study included 278 rectal cancer patients, comprising 179 males and 99 females, ranging in age from 24 to 90 years. Among these patients, 128 had wild‐type *KRAS*, and 150 had mutations in *KRAS*. As shown in Table 1, a statistically significant difference was observed in lymph node enlargement between *KRAS* wild‐type and mutant groups in the training set (*p* = 0.009), and in the test set, a significant difference was found in maximum tumor diameter between the two groups (*p* = 0.039). No significant differences were found in other baseline characteristics, including gender, age, tumor thickness, distance from the anal margin, CA199 and CEA levels, and pathological T stage.

**TABLE 1:** Baseline characteristics of patients in the training and test groups.

Training group				Test group		
	Wide‐type	Mutant type	*p value*	Wide‐type	Mutant type	*p value*
	*n* = 89	*n* = 105		*n* = 39	*n* = 45	
Age/Year						
<65	56(62.92)	55(52.38)	0.183	26(66.67)	27(60.00)	0.686
≥65	33(37.08)	50(47.62)		13(33.33)	18(40.00)	
Sex						
Male	61(68.54)	63(60.00)	0.278	26(66.67)	29(64.44)	1
Female	28(31.46)	42(40.00)		13(33.33)	16(35.56)	
T stage						
T1	9(10.11)	3(2.86)	0.091	3(7.69)	1(2.22)	0.69
T2	10(11.24)	21(20.00)		10(25.64)	11(24.44)	
T3	52(58.43)	59(56.19)		17(43.59)	22(48.89)	
T4	18(20.22)	22(20.95)		9(23.08)	11(24.44)	
CEA/(ng·mL^−1^)						
≤5	55(61.80)	64(60.95)	1	25(64.10)	28(62.22)	1
>5	34(38.20)	41(39.05)		14(35.90)	17(37.78)	
Thickness/cm	1.53 ± 0.57	1.59 ± 0.62	0.486	1.43 ± 0.61	1.49 ± 0.44	0.15
Maximum diameter/cm	4.27 ± 1.86	3.85 ± 1.55	0.13	3.64 ± 1.76	4.21 ± 1.26	0.039
CA199/(U·mL^−1^)						
<37	78(87.64)	88(83.81)	0.581	34(87.18)	38(84.44)	0.964
≥37	11(12.36)	17(16.19)		5(12.82)	7(15.56)	
Distance from the anal margin/cm						
≤5	24(26.97)	41(39.05)	0.206	8(20.51)	14(31.11)	0.466
5–10	52(58.43)	51(48.57)		23(58.97)	21(46.67)	
>10	13(14.61)	13(12.38)		8(20.51)	10(22.22)	
Lymph node enlargement						
Yes	55(61.80)	44(41.90)	0.009	20(51.28)	26(57.78)	0.706
NO	34(38.20)	61(58.10)		19(48.72)	19(42.22)	

### 3.2 Feature selection

**FIGURE 2:**
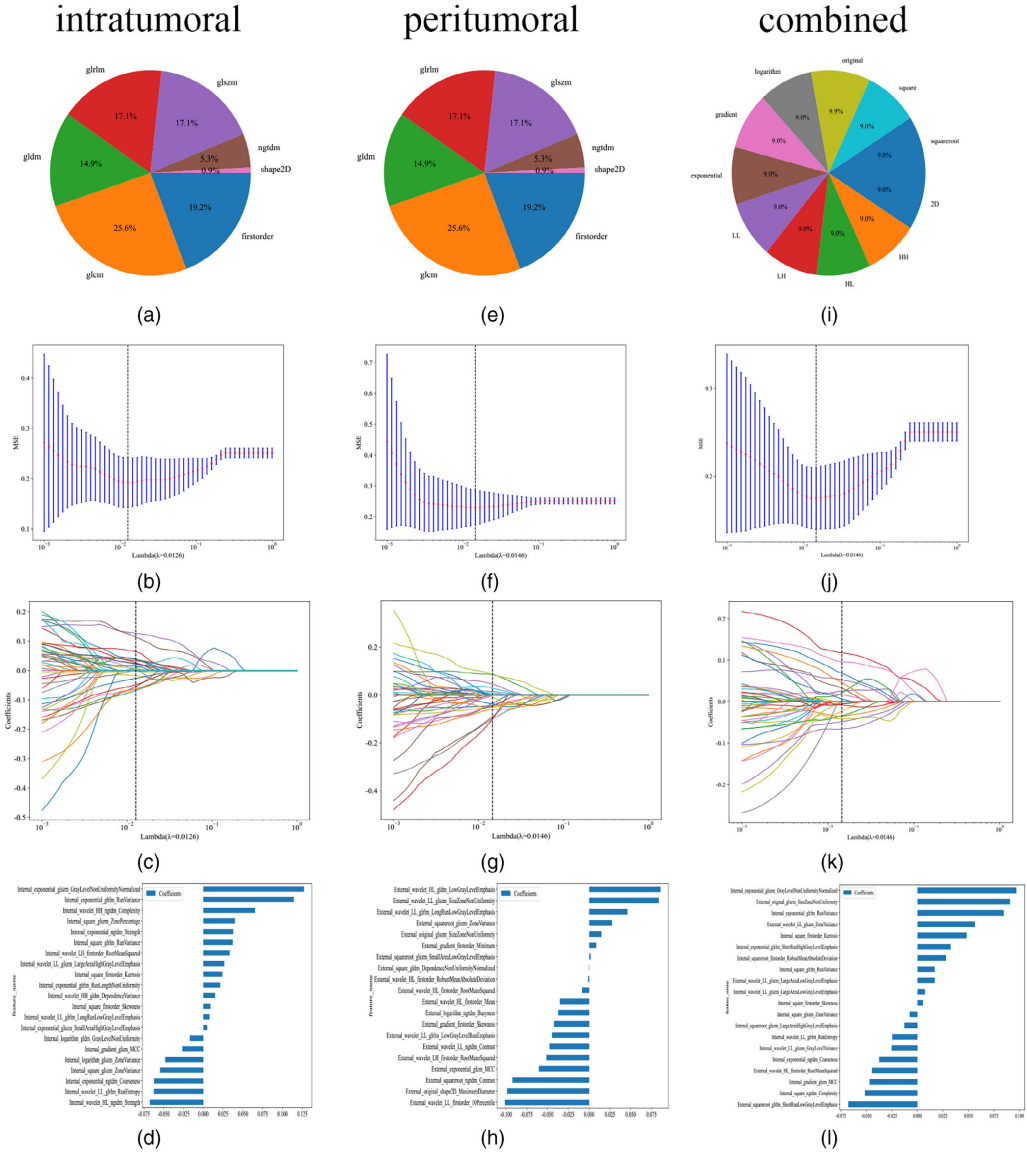
(1) Distribution and proportion of various radiomics features. Intratumoral (a); peritumoral (e); and combined (i). (2) Radiomic features selection based on least absolute shrinkage and selection operator algorithm. Intratumoral (b, c); peritumoral (f, g); and combined (j, k). (3) Histogram of the Rad‐score based on the selected features. Intratumoral (d); peritumoral (h); and combined (l).

For each patient, 1032 intratumoral and peritumoral radiomics features were extracted. Following feature screening, 21 and 20 features were selected for constructing the intratumoral and peritumoral prediction models, respectively. Subsequently, all features were merged into 2064 radiomics features through early fusion. Ultimately, 22 features were selected to construct the combined model (Figure 2). A five‐fold cross‐validated grid search was performed on the training set to identify the optimal hyperparameters for the LightGBM model: max_depth = 3 and n_estimators = 10.

### 3.3 Performance and validation of predictive models

Table 2 presents the performance metrics of all models on both the training and test sets, including accuracy, area under the curve (AUC), sensitivity, and specificity.

**TABLE 2:** Evaluation results of the models in the training and test groups.

Models	Group	Accuracy	AUC	95% CI	Sensitivity	Specificity	PPV	NPV	*p* value	*p* value
Internal_LightGBM	Train	0.83	0.928	0.895–0.961	0.743	0.933	0.929	0.755	Reference	0.046
Internal_LR	Train	0.825	0.887	0.842–0.932	0.857	0.787	0.826	0.824	0.046	Reference
Internal_SVM	Train	0.856	0.912	0.871– 0.953	0.848	0.865	0.881	0.828	0.379	0.012
Internal_LightGBM	Test	0.714	0.76	0.657– 0.863	0.6	0.846	0.818	0.647	Reference	0.509
Internal_LR	Test	0.726	0.795	0.702– 0.889	0.756	0.692	0.739	0.711	0.509	Reference
Internal_SVM	Test	0.75	0.799	0.704– 0.894	0.733	0.769	0.786	0.714	0.464	0.894
External_LightGBM	Train	0.856	0.924	0.889– 0.960	0.886	0.82	0.853	0.859	Reference	0.001
External_LR	Train	0.773	0.837	0.779– 0.895	0.667	0.899	0.886	0.696	0.001	Reference
External_SVM	Train	0.84	0.907	0.865– 0.949	0.81	0.876	0.885	0.796	0.4	0
External_LightGBM	Test	0.631	0.642	0.523– 0.761	0.422	0.872	0.792	0.567	Reference	0.323
External_LR	Test	0.595	0.584	0.458– 0.710	0.667	0.513	0.612	0.571	0.323	Reference
External_SVM	Test	0.607	0.618	0.497– 0.739	0.356	0.897	0.8	0.547	0.64	0.487
Combine_LightGBM	Train	0.851	0.925	0.891– 0.960	0.924	0.764	0.822	0.895	Reference	0.016
Combine_LR	Train	0.794	0.882	0.837– 0.928	0.762	0.831	0.842	0.747	0.016	Reference
Combine_SVM	Train	0.809	0.892	0.847– 0.937	0.676	0.966	0.959	0.717	0.05	0.387
Combine_LightGBM	Test	0.821	0.894	0.825– 0.963	0.911	0.718	0.788	0.875	Reference	0.758
Combine_LR	Test	0.833	0.881	0.808– 0.955	0.911	0.744	0.804	0.879	0.758	Reference
Combine_SVM	Test	0.798	0.889	0.820– 0.958	0.733	0.872	0.868	0.739	0.88	0.808

*p* value was calculated by Delong test.

**FIGURE 3:**
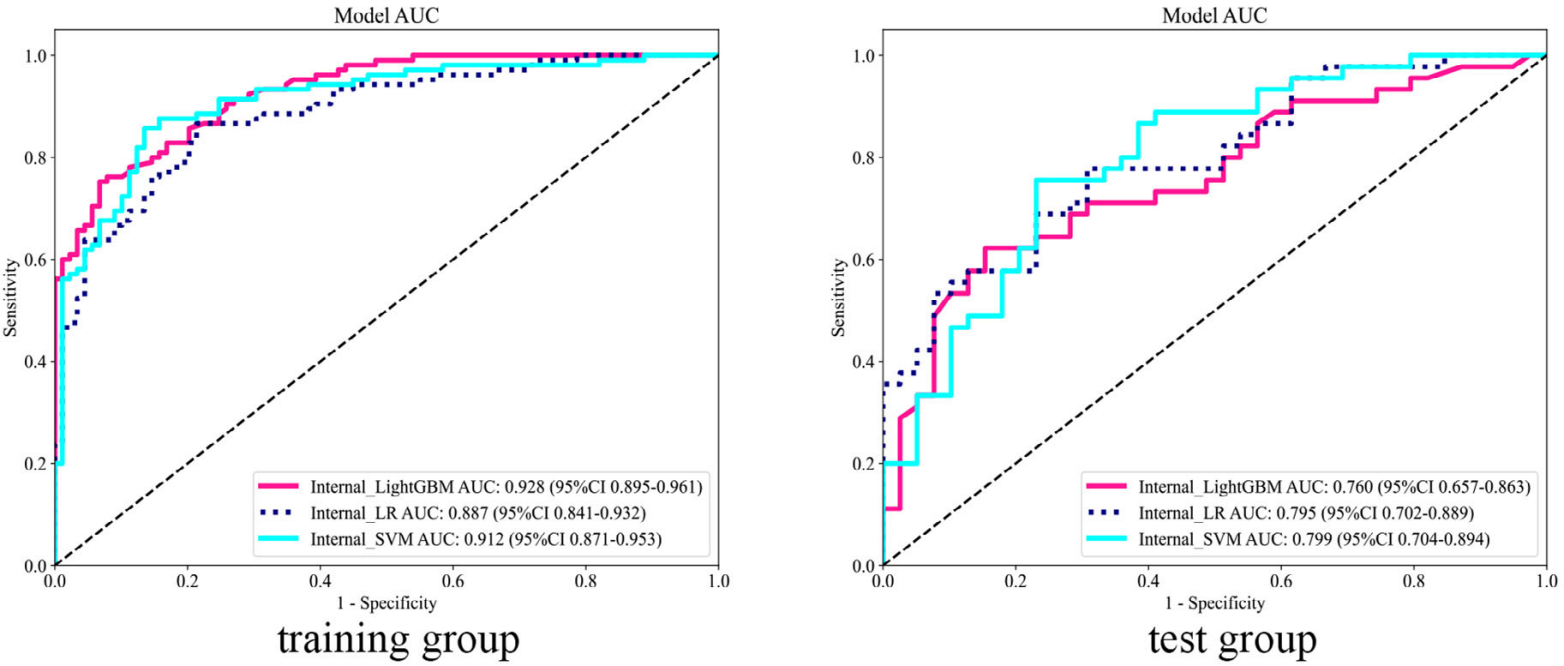
Receiver operating characteristic curves of the intratumoral model for the training and test groups.

**FIGURE 4:**
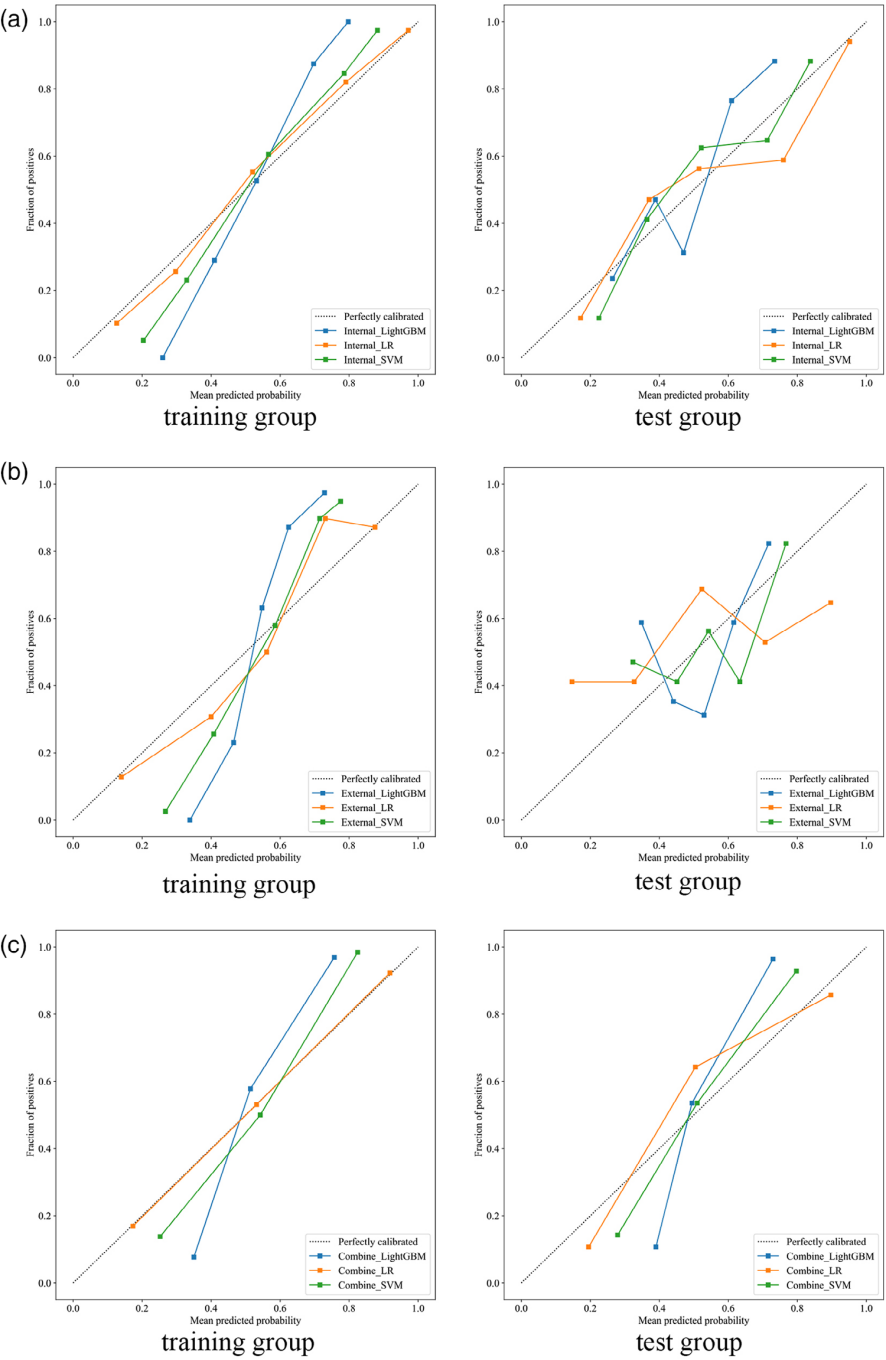
Calibration curve of the different models in the training and test group. (a) Calibration curve of different intratumoral models. (b) Calibration curve of different peritumoral models. (c) Calibration curve of different combined models.

**FIGURE 5:**
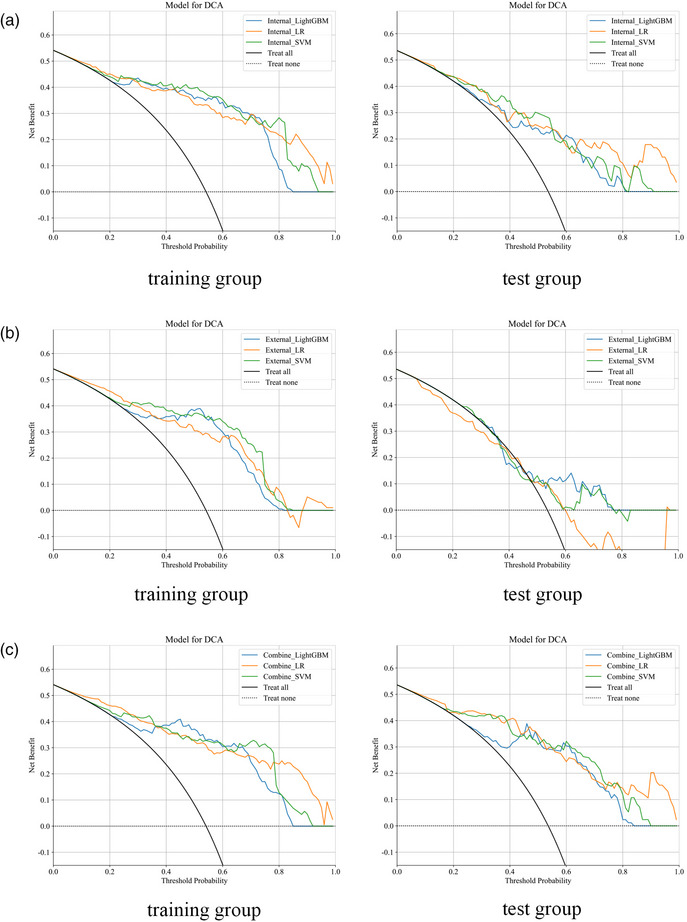
Decision curve analysis (DCA) of the different models in the training and test group. (a) DCA of different intratumoral models. (b) DCA of different peritumoral models. (c) DCA of different combined models.

In the intratumoral model, although the Internal_SVM model had the highest AUC (0.799) in the test set (Figure 3), the DeLong test indicated no significant difference compared to other models (*p *> 0.05), and all calibration curves demonstrated good consistency (Figure 4). Therefore, we consider the Internal_LR model, with an AUC of 0.795, which shows the best clinical net benefit on the DCA (Figure 5), to be the most effective.

**FIGURE 6:**
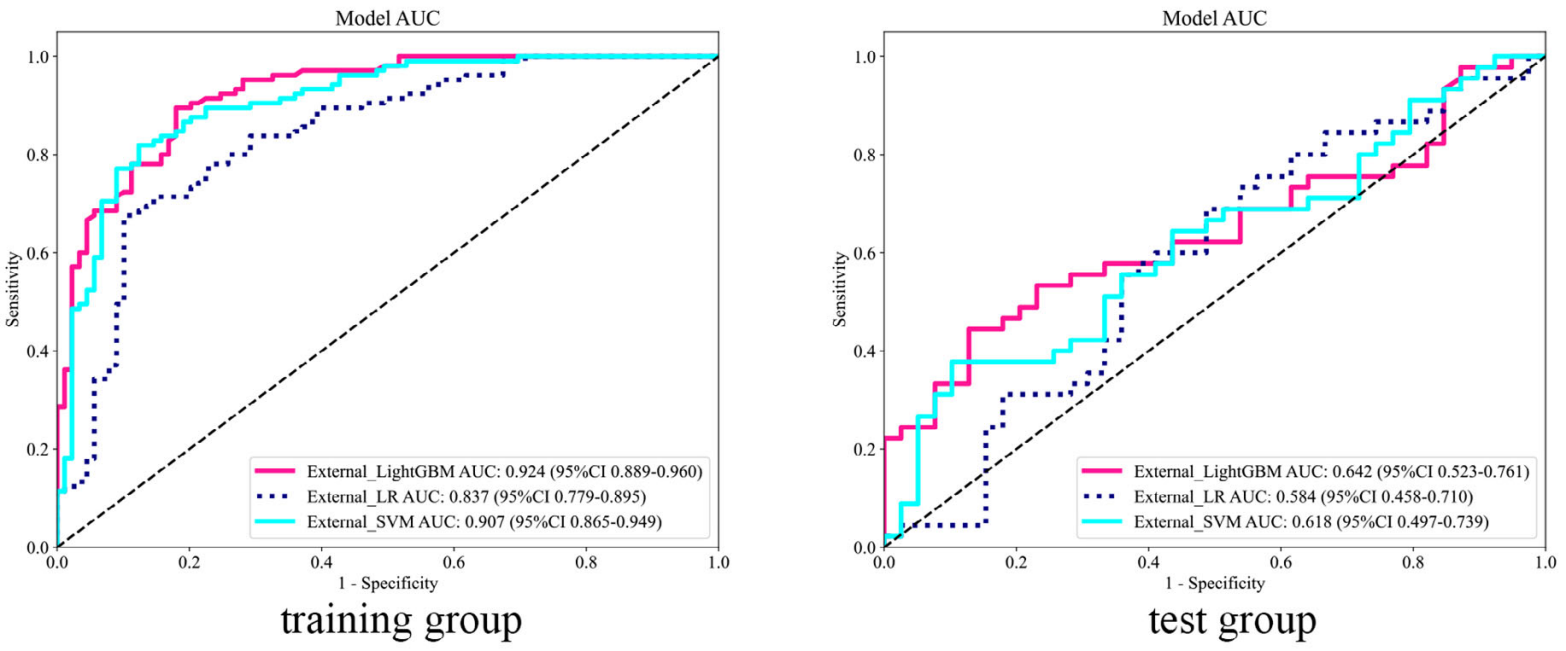
Receiver operating characteristic curves of the peritumoral model for the training and test groups.

In the peritumoral model, the External_LightGBM model had the highest AUC (0.642) in the test set and showed the greatest clinical net benefit on the DCA (Figures 5 and 6). However, its calibration curve exhibited poor consistency. Therefore, we consider the External_SVM model, which exhibited better calibration (AUC = 0.618), to be the most effective (Figure 4).

**FIGURE 7:**
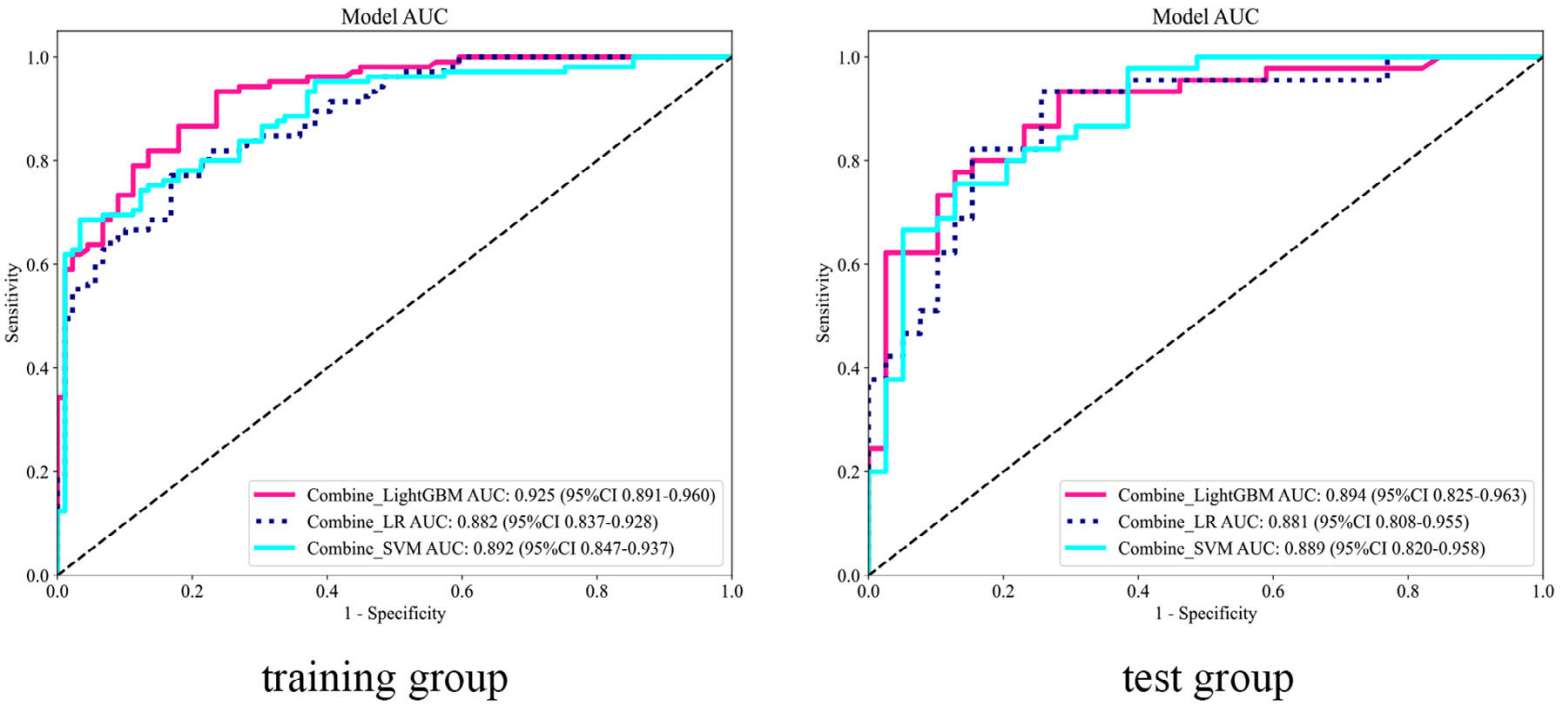
Receiver operating characteristic curves of the combined models for the training and test groups.

In the combined model, although the AUC of the Combine_LR model was the lowest on both the training and test sets, at 0.882 and 0.881 (Figure 7), respectively, the DeLong test showed no significant difference compared to other models on the test set (*p *> 0.05). Additionally, the DCA indicated that its clinical net benefit was not significantly different from that of other models (Figure 5). The calibration curve of the Combine_LR model demonstrated good consistency, unlike the other models with poorer consistency (Figure 4). Therefore, we consider this model to be the most effective.

**FIGURE 8:**
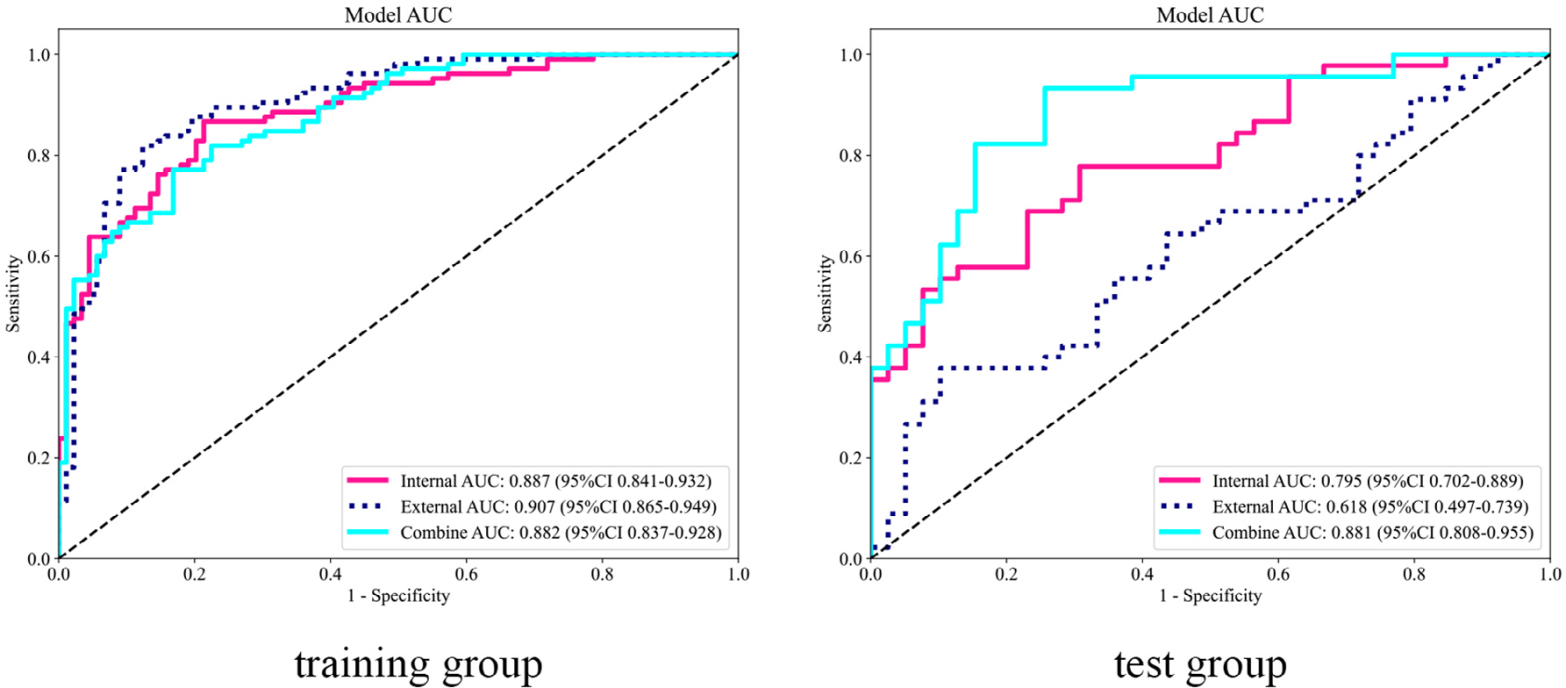
Receiver operating characteristic curves of the optimal intratumoral, peritumoral, and combined models for the training and test groups.

**FIGURE 9:**
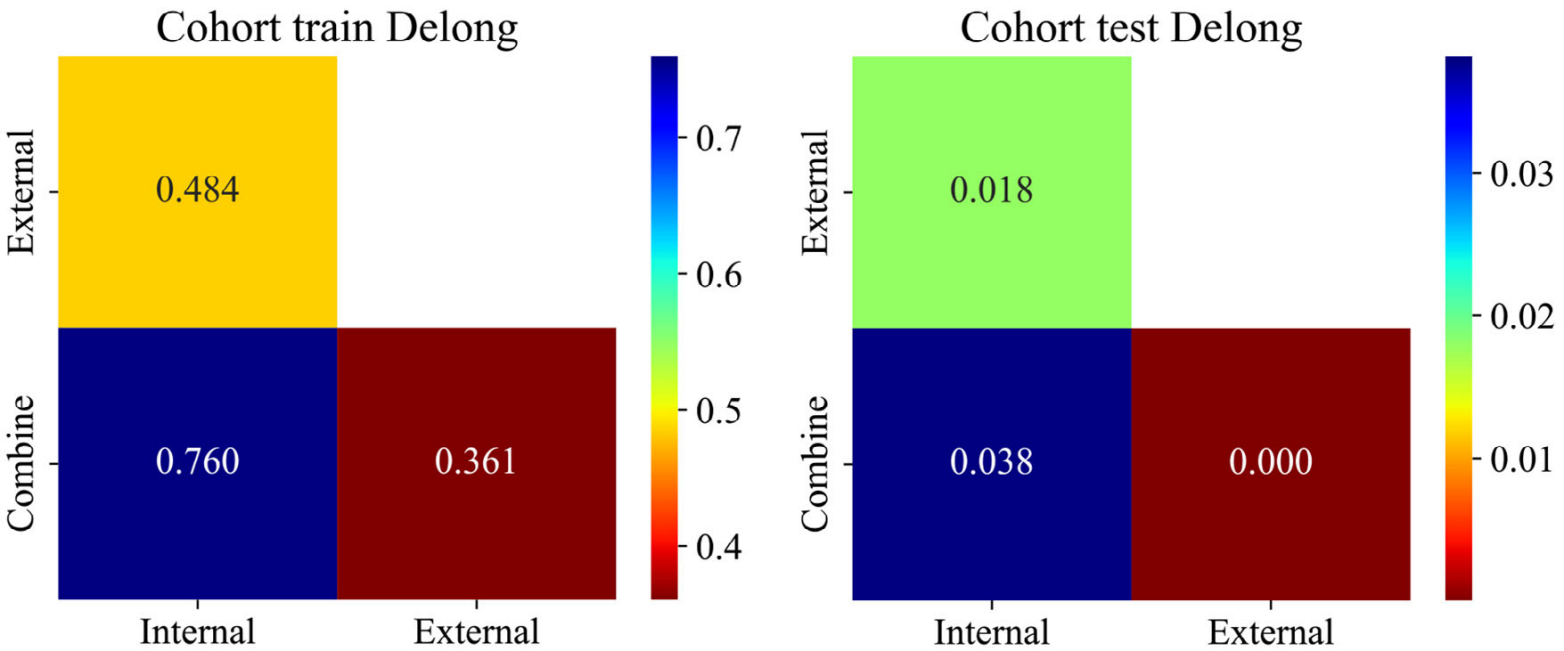
DeLong test results for the optimal intratumoral, peritumoral, and combined models in the training and test groups.

**FIGURE 10:**
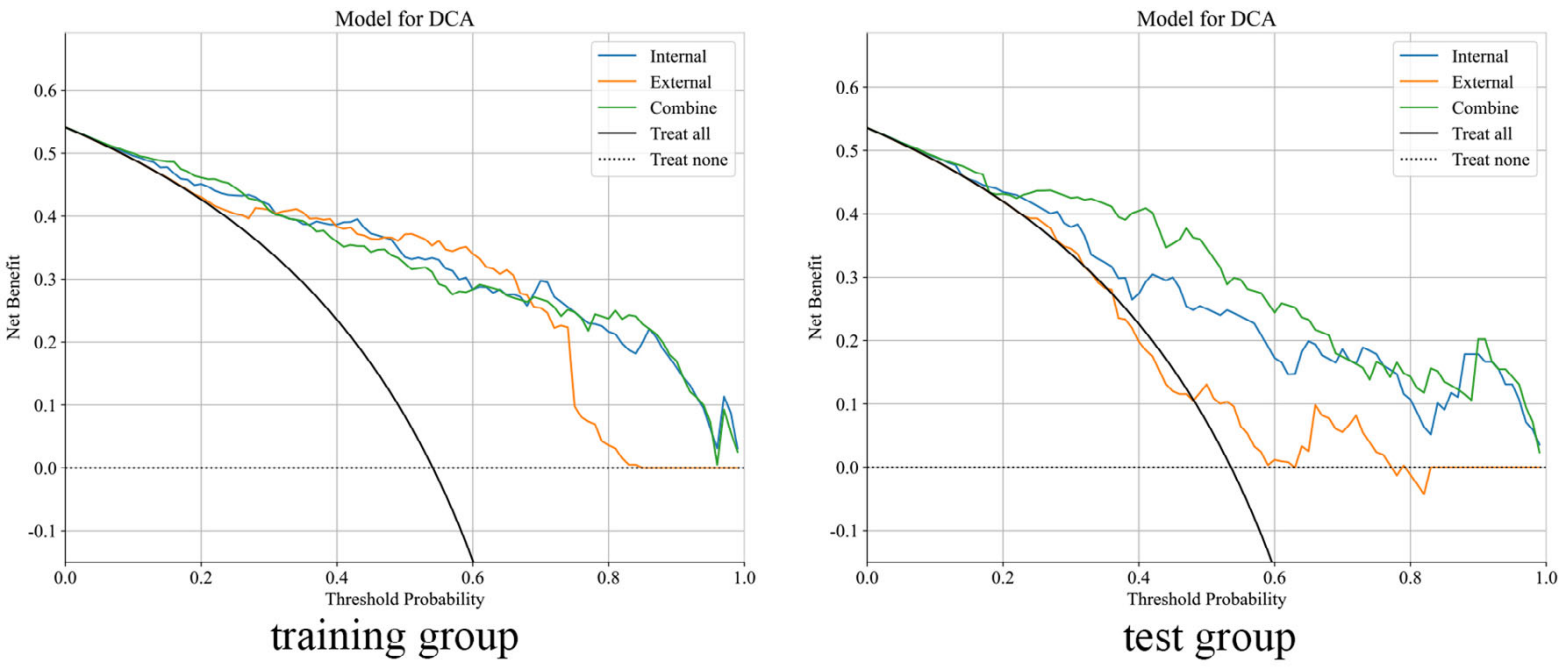
Decision curve analysis of the optimal intratumoral, peritumoral, and combined models for the training and test groups.

As shown in Figure 8, the combined model achieves the highest AUC compared to the intratumoral model or the peritumoral model alone. Furthermore, the DeLong test indicates a significant difference between the combined model and the others (*p* < 0.05) (Figure 9). The DCA demonstrates that the combined model provides significant clinical benefits (Figure 10). This suggests that the peritumoral region can provide Supporting Information for prediction.

### 3.4 Generalizability of the models

We conducted subgroup analysis based on lymph node enlargement (LN+ vs. LN−), a variable exhibiting statistically significant differences between *KRAS* wild‐type and mutant groups in the training set (*p* = 0.009). The model demonstrated comparable predictive performance in both subgroups, with overlapping 95% confidence intervals of AUC values, indicating consistent discriminative capability and potential applicability across different clinical scenarios (Table 3). Additionally, maximum tumor diameter differed significantly between *KRAS* wild‐type and mutant groups in the test set (*P* = 0.039). Although we did not conduct a dedicated subgroup analysis for tumor diameter due to its continuous nature and limited subgroup size, we recognize it as a potential confounding factor that might influence predictive performance.

**TABLE 3:** Evaluation results of the lymph node subtgroup models in the test group.

Models	Accuracy	AUC	95% CI	*p* value	*p* value
Internal_LN+_LR	0.739	0.781	0.648–0.914	Reference	1
Internal_LN+_SVM	0.739	0.781	0.643–0.919	1	Reference
Internal_LN+_LightGBM	0.674	0.739	0.596–0.883	0.56	0.612
Internal_LN‐_LR	0.737	0.809	0.672–0.946	Reference	0.841
Internal_LN‐_SVM	0.737	0.817	0.682–0.953	0.841	Reference
Internal_LN‐_LightGBM	0.763	0.798	0.650–0.946	0.895	0.792
External_LN+_LR	0.609	0.575	0.399– 0.751	Reference	0.624
External_LN+_SVM	0.609	0.61	0.443–0.776	0.624	Reference
External_LN+_LightGBM	0.565	0.608	0.442–0.773	0.68	0.978
External_LN‐_LR	0.605	0.615	0.430–0.800	Reference	0.582
External_LN‐_SVM	0.658	0.654	0.474–0.834	0.582	Reference
External_LN‐_LightGBM	0.684	0.712	0.539– 0.885	0.269	0.455
Combine_LN+_LR	0.848	0.896	0.801–0.991	Reference	0.627
Combine_LN+_SVM	0.826	0.917	0.842– 0.993	0.627	Reference
Combine_LN+_LightGBM	0.848	0.917	0.834–1.000	0.696	1
Combine_LN‐_LR	0.789	0.859	0.738– 0.980	Reference	0.903
Combine_LN‐_SVM	0.789	0.864	0.746– 0.983	0.903	Reference
Combine_LN‐_LightGBM	0.763	0.87	0.756– 0.984	0.851	0.913

*p* value was calculated by Delong test.

### 3.5 Interpretation of the models

**FIGURE 11:**
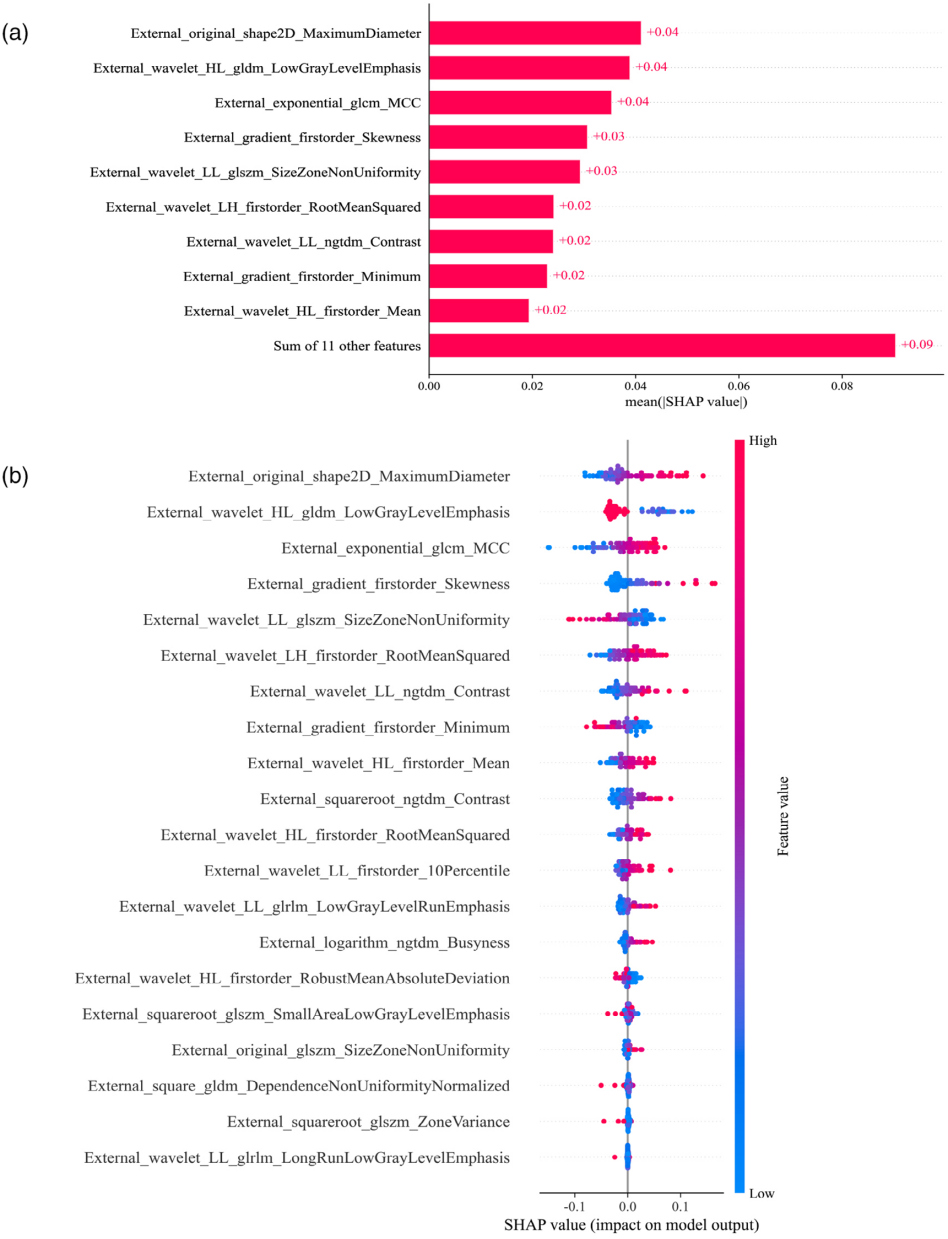
SHapley Additive explanation (SHAP) analysis of the External_SVM model for predicting *KRAS* mutations in rectal tumors. (a) shows the feature importance ranking determined by the mean absolute SHAP values. (b) presents a summary plot with SHAP values, offering a comprehensive visualization of the cumulative impact of each feature.

**FIGURE 12:**
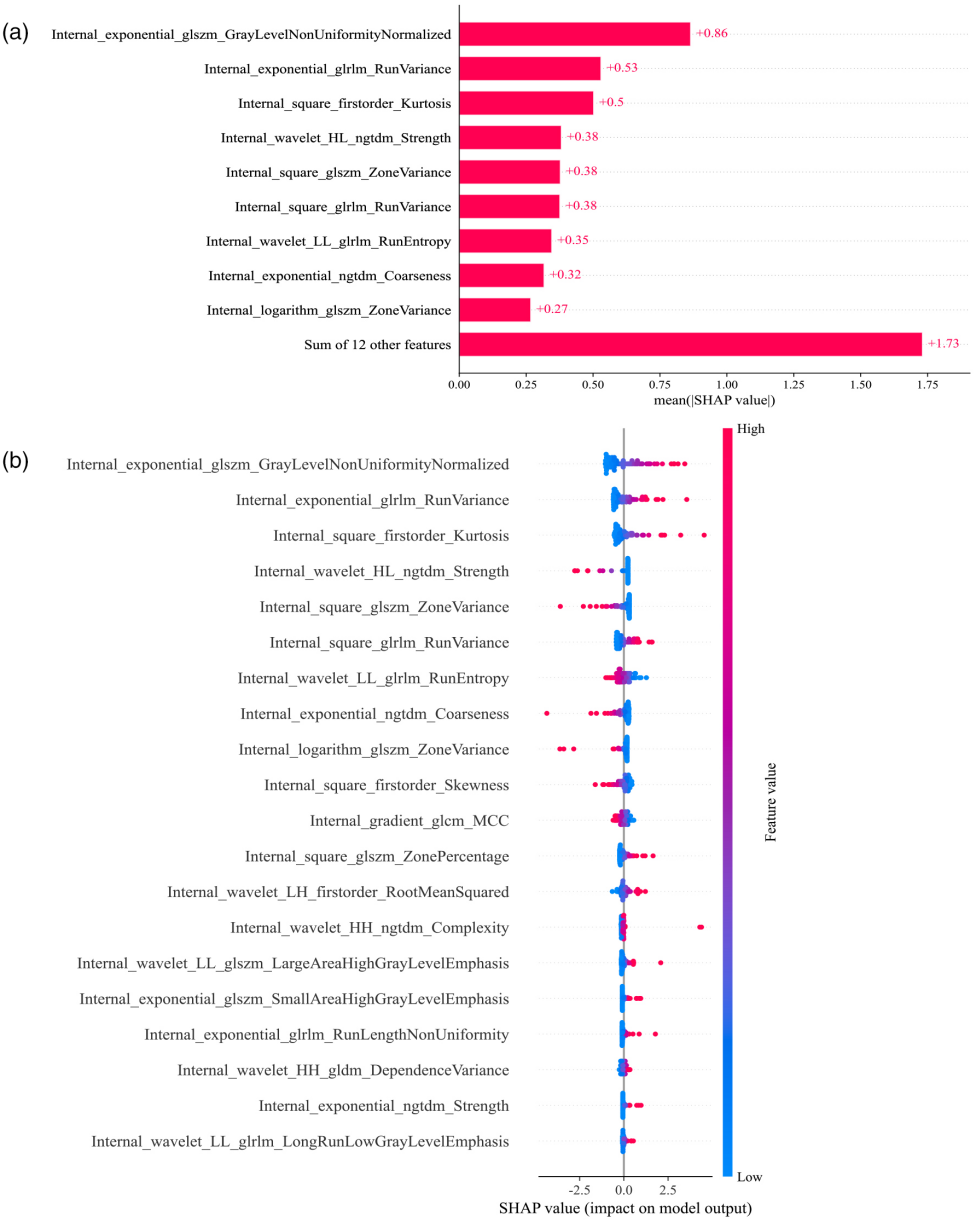
SHapley Additive explanation (SHAP) analysis of the Internal_LR model for predicting *KRAS* mutations in rectal tumors. (a) shows the feature importance ranking determined by the mean absolute SHAP values. (b) presents a summary plot with SHAP values, offering a comprehensive visualization of the cumulative impact of each feature.

**FIGURE 13:**
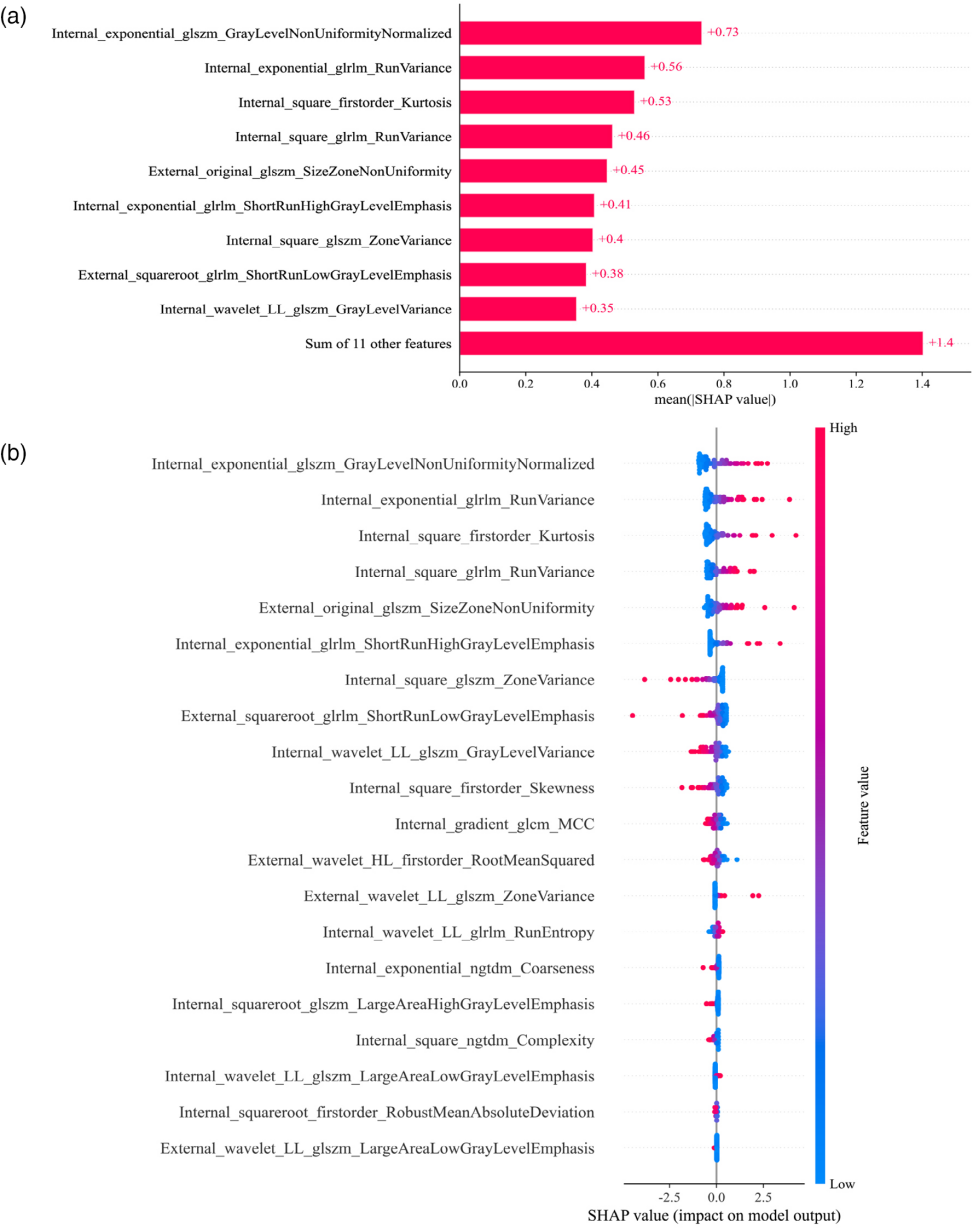
SHapley Additive explanation (SHAP) analysis of the Combine_LR model for predicting *KRAS* mutations in rectal tumors. (a) shows the feature importance ranking determined by the mean absolute SHAP values. (b) presents a summary plot with SHAP values, offering a comprehensive visualization of the cumulative impact of each feature.

The SHAP analysis was employed to interpret the Internal_LR, External_SVM, and Combine_LR models, allowing for the quantification of the influence of each feature. By calculating the absolute average SHAP values, it facilitates prioritizing features based on their importance. It is worth noting that in the External_SVM model, no radiomic feature emerged as an exceptionally important factor (Figure 11a). However, Internal_exponential_glszm_GrayLevelNonUniformityNormalized was the most influential factor in the Internal_LR and Combine_LR models (Figures 12a and 13a). Figures 11, 12, and 13b summarize the collective impact of these features, described by their SHAP values. This visualization provides a comprehensive understanding of how each feature contributes to predicting *KRAS* mutation status in individual patients. Importantly, higher values of this feature correspond to a greater likelihood of *KRAS* mutation.

## 4 DISCUSSION

Early detection of *KRAS* mutation status in rectal cancer is crucial for selecting appropriate clinical targeted therapies. In our study, we developed and validated a combined prediction model that incorporates both intratumoral and peritumoral features. This model demonstrated superior prediction efficiency and clinical benefit in the test group, outperforming the models based solely on intratumoral or peritumoral features.

Previous research has revealed that the tumor microenvironment comprises various immune cells, blood vessels, and extracellular matrix components.^25^ Alterations in the distribution of immune cells and angiogenesis can facilitate tumor development and metastasis. By analyzing imaging features of the tissues surrounding the tumor, we can gain insights into the heterogeneity and complexity of the tumor microenvironment, aiding in the evaluation of its biological behavior and the implementation of early interventions.^26^ In the realm of rectal cancer research, numerous studies have established associations between peritumoral radiological features and various clinical outcomes, such as response to neoadjuvant therapy, lymph node metastasis, nerve invasion, disease‐free survival, and overall survival.^27^, ^28^, ^29^, ^30^ These studies validate the feasibility of our research approach. Our study discovered that the peritumoral tissue of rectal cancer plays a significant role in predicting *KRAS* mutations. This may be linked to the enhanced malignant proliferation of cells following *KRAS* mutation, along with invasion and infiltration into the peritumoral area, resulting in increased complexity of the peritumoral tissue composition.

Incorporating SHAP analysis enhanced the interpretability of the Internal_LR and combined_LR models, emphasizing that the ecponential_glszm_GrayLevelNonUniformityNormalized feature in the two models has the greatest impact on predicting the outcomes for specific samples, and contributes the most to the prediction across the entire dataset. The GrayLevelNonUniformityNormalized feature belongs to texture features, measuring the variability of the gray intensity value in the ROI. A higher value is associated with a higher probability of *KRAS* mutation. The disparity in this feature between *KRAS* mutant and wild‐type lesions may be associated with the malignant proliferation, decreased apoptosis of cells, and complex tissue composition following *KRAS* mutation.

In this study, ROI segmentation was concurrently performed by two experienced ultrasound doctors, and the first doctor re‐segmented the ROI after some time. Only features with an intraclass correlation coefficient >0.75 were retained for further analysis, ensuring feature stability at the segmentation level. Feature extraction was conducted using the Pyradiomics software, which has excellent reliability across all features.^31^ Previous research on the reproducibility of ultrasound omics features has indicated that wavelet and shear wave characteristics exhibit the highest consistency.^32^ The relatively low proportion of wavelet features selected in this study may be attributed to heterogeneity among different cancer types. Future research will delve deeper into the predictive value of shear wave features for the *KRAS* in rectal cancer, aiming to more comprehensively evaluate the efficacy of ultrasound in detecting *KRAS* mutations in rectal cancer.

Currently, the application of radiomics in predicting *KRAS* mutations in rectal cancer has been explored in several studies. For instance, He et al.^33^ reported an accuracy of 68.2% using 18F‐FDG PET/CT for predicting *KRAS* mutations in colorectal cancer, significantly lower than the accuracy achieved in our study. Although CT scans are widely used clinically, they carry radiation risks and have limited capability to clearly visualize the rectal wall's layered structure. Li et al.^34^ evaluated the predictive value of CT‐based radiomics features for *KRAS* mutations, obtaining an AUC of 0.813, which remains below the performance demonstrated in this study. MRI, which is radiation‐free and excels in soft tissue visualization, was employed by Liu et al.^35^ to develop a deep learning model combining MRI features and clinical data, reaching an AUC of 0.84. However, these studies primarily focused on intratumoral characteristics, neglecting potentially informative peritumoral features—a common limitation in prior research.

ERUS is user‐friendly and free from radiation hazards. The high frequency intrarectal ultrasound probe effectively delineates the rectal wall's layered structure. Our research encompasses features within and around the tumor. The test group's AUC was 88.9%, surpassing that of previous studies. This indicates that our model has superior predictive efficiency and offers distinct advantages.

In addition, a previous ERUS‐based study incorporated both radiomics and deep learning features across various peritumoral ranges (10, 20, and 30 pixels) and achieved a best AUC of 0.896 through feature fusion. In contrast, the present study adopted a fixed 5 mm anatomical peritumoral region and used radiomics features alone, achieving a comparable AUC of 0.889. This suggests that physically standardized peritumoral extensions may offer favorable reproducibility without compromising predictive performance. However, this study did not examine the influence of different peritumoral extension widths on model performance. The potential impact of varying peritumoral ranges warrants further investigation in future studies. Furthermore, subgroup analysis based on lymph node enlargement was introduced to evaluate generalizability across different patient subgroups—an aspect not addressed in the previous work.

This study has some limitations. First, being retrospective, it may contain selection bias. Future studies should include multicenter data for external validation to reduce this bias. Second, although ICC filtering was applied to select stable radiomic features, manual segmentation may still introduce interobserver variability. Future studies may consider incorporating automatic segmentation methods to further improve consistency. Third, while subgroup analysis based on lymph node enlargement was performed to account for clinical heterogeneity, tumor diameter—being a continuous variable and limited by subgroup size—was not analyzed separately. This may represent a potential confounding factor influencing model performance and should be addressed in future research. Finally, this study did not compare different feature extraction and selection strategies; thus, the final extracted features might not be the most optimal, potentially impacting the model's predictive performance. Future work could explore and compare alternative methods to improve robustness.

In conclusion, the ultrasound radiomics model, utilizing both intratumoral and peritumoral features, demonstrates effective diagnostic capability in predicting *KRAS* status in rectal cancer patients. These findings highlight the clinical value of using physically defined peritumoral regions in ultrasound radiomics and support the model's potential as a noninvasive tool for guiding targeted therapy decision.

## 5 CONCLUSIONS

The ultrasound radiomics model, utilizing both intratumoral and peritumoral features, demonstrates effective diagnostic capability in predicting *KRAS* status in rectal cancer patients. This model holds potential to aid in guiding the selection of clinical targeted therapies.

## AUTHOR CONTRIBUTIONS

Yajiao Gan and Zhikui Chen conceived and designed the project. Mingling Zhuo, Qingfu Qian, and Qingling Shen collected the data. Peng Lin and Ensheng Xue analyzed and interpreted the data. Yajiao Gan and Qiping Hu drafted the manuscript. All the authors edited and made critical revisions to the article. All authors read and approved the final manuscript.

## CONFLICT OF INTEREST STATEMENT

The authors have no relevant financial or nonfinancial interests to disclose.

## ETHICS APPROVAL

This study was approved by the Medical Ethics Committee of Fujian Medical University Union Hospital (Approval No.: 2023KY082), and informed consent from the subjects was not required.
